# Case report: Amnestic mild cognitive impairment in multiple domains associated with neurofascin 186 autoantibodies: Case series with follow-up and review

**DOI:** 10.3389/fpsyt.2022.1054461

**Published:** 2023-01-12

**Authors:** Niels Hansen, Anne Sagebiel, Kristin Rentzsch, Sina Hirschel, Jens Wiltfang, Björn H. Schott, Bartels Claudia

**Affiliations:** ^1^Department of Psychiatry and Psychotherapy, University Medical Center Göttingen, Göttingen, Germany; ^2^Department of Psychiatry and Psychotherapy, Translational Psychoneuroscience, University Medical Center Göttingen, Göttingen, Germany; ^3^Clinical Immunological Laboratory Prof. Stöcker, Groß Grönau, Germany; ^4^German Center for Neurodegenerative Diseases (DZNE), Göttingen, Germany; ^5^Department of Medical Sciences, Neurosciences and Signaling Group, Institute of Biomedicine (iBiMED), University of Aveiro, Aveiro, Portugal; ^6^Leibniz Institute for Neurobiology, University of Magdeburg, Magdeburg, Germany

**Keywords:** autoantibody, autoimmunity, cognition, neurofascin 186 antibody, mild cognitive impairment

## Abstract

**Background:**

Neurofascin 186 autoantibodies are known to occur with a diseased peripheral nervous system. Recently, also additional central nervous system (CNS) involvement has been reported in conjunction with neurofascin 186 autoantibodies. Our case enlarges the spectrum of neurofascin 186 antibody-related disease to include mild cognitive impairment (MCI).

**Methods:**

We report here a case after having examined the patient files retrospectively, including diagnostics such as blood and cerebrospinal fluid (CSF) analysis involving the determination of neural autoantibodies, brain magnetic resonance imaging (MRI), brain fluorodesoxyglucose positron emission tomography (FDG-PET), and extensive neuropsychological testing.

**Results:**

We report on two patients with MCI. Brain MRI showed cerebral microangiopathy in both patients, but brain FDG-PET demonstrated pathology in the right prefrontal cortex, in the right inferior parietal cortex, and in both lateral occipital cortices in one patient. Neurofascin 186 antibodies were detected in serum in both patients, and neurofascin 186 autoantibodies were also detected in the CSF of one of these patients. At follow-up six month later, neurofascin 186 autoantibodies disappeared in one patient while persisting in the other.

**Conclusion:**

We report on two individuals presenting MCI associated with neurofascin 186 antibodies, thus expanding the potential spectrum of neurofascin 186-associated disease. This report supports the recommendation to consider also neurofascin 186 autoantibodies in not just peripheral nerve disease, but also in disorders involving CNS autoimmunity. More studies are needed to clarify the lack of association between neurofascin 186 autoantibodies and cognitive decline.

## 1. Introduction

The spectrum of the diseases associated with neurofascin 186 antibodies is limited mainly to peripheral neuropathy (1) such as chronic demyelinating inflammatory polyneuropathy (2) as well as subacute nodopathy (3) and to amyotrophic lateral sclerosis (4). Recently, also central nervous system (CNS) involvement in neurofascin 186 autoantibody-associated peripheral neuropathy has been demonstrated (5). However, cognitive impairment has been rarely reported in association with neurofascin 186 antibodies (5), and there might be structures in the CNS involved in primary neuroinflammation in the peripheral nervous system. Here we report two patients predominantly presenting with cognitive impairment as a clinical phenotype in which primary neuroinflammatory locus is probably not the peripheral nervous system. Our report thus highlights the novelty of a neurofascin 186 autoantibody-related affectation of the CNS through a possible inflammatory process associated with cognitive impairment.

## 2. Case reports

### 2.1. Case 1

**FIGURE 1:**
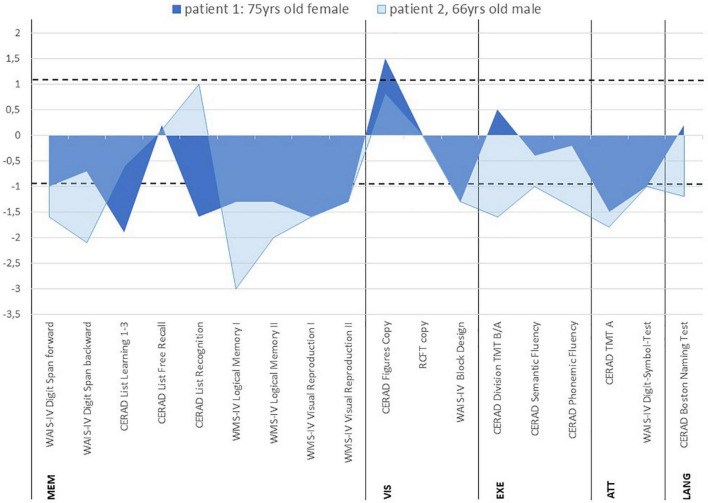
Neuropsychological profiles of patients with anti-neurofascin 186 autoantibody-associated cognitive impairment *z*-scores. The area between dotted lines denote the normal range. CERAD, consortium to establish a registry for Alzheimer’s disease; WMS IV, wechsler memory scale – 4^th^ edition; RCFT, rey-osterrieth complex figure test; WAIS-IV, wechsler adult intelligence scale – 4^th^ edition; TMT, trail making test; MEM, memory; VIS, visuospatial cognition; EXE, executive functions; ATT, attention; LANG, language.

A 75-year-old woman presented complaining of short-term memory disturbances, word-finding difficulties and depressive symptoms starting about a year earlier. She is a multimorbid patient with high care needs. Her comorbidities comprised essential tremor, lung empyema, steatosis hepatis with multiple liver cysts, cholecystolithiasis, coronary heart disease, arterial hypertension, mitral valve insufficiency, hyperlipoproteinemia, hypothyreosis, and polyneuropathy. She also has a knee total endoprothesis on the left and coxathrosis on the right. She also has about 60 pack years involving nicotine abuse, but has probably been abstinent since 2019. Her mother suffered from dementia. She has been categorized as care level two and is about 70% disabled (i.e., severely disabled). Her daughter serves as a complete health care proxy for her. She acquired a secondary school level 1 certificate and worked as a telephone assistant in telecommunications. Concurrent mediation comprised the following: pantoprazole 20 mg/d, atorvastatin 20 mg/d, L-thyroxin 50 mikrog/d, fexofenadin 180 mg/d and bupropion 150 mg/d. Psychopathological examination revealed a loss of drive and a slowed psychomotor speed. Furthermore, she was also suffering from depression (ICD-10: F33.1: recurrent major depression, moderate, Geriatric Depression Scale (GDS) score: 6, i.e., depressive symptoms of mild to moderate severity). Neurological examination demonstrated pallanesthesia in her legs. At cognitive screening, she scored 29 of 30 points on the Mini Mental Status Examination (MMST), but neuropsychological testing revealed an amnestic mild cognitive impairment (MCI) with deficits in information processing speed (attention), visuospatial cognition, verbal and figural memory (Figure 1). Together with cognitive deterioration in multiple domains, a caregiver rating (Bayer Activities of Daily Living Scale, B-ADL) resulted in mild impairments of ADL competence (mean B-ADL: 3.3). Magnetic resonance imaging (MRI) revealed cerebral microangiopathy with Fazekas grade 1. Cerebrospinal fluid (CSF) analysis showed elevated S100 protein (4.7 μg/l, pathological if > 2.7 μg/l) (Table 1) and ptau 181 protein (Table 1). We also identified intrathecal IgG synthesis (Table 1). We also detected anti-neurofascin 186 autoantibodies in serum at 1:32 intensity via anti-neural antigen IgG immunofluorescence testing with BIOCHIP-mosaic with brain tissue and recombinant cells. At follow-up six months later, no serum neurofascin 186 autoantibodies were detectable. Her familiar MCI was evident over the course, but dementia-like syndrome was not.

**TABLE 1:** Neurofascin 186 autoantibodies in neuropsychiatric disease.

Neuropsychiatric disease	References
Amyotrophic lateral sclerosis	(4)
Anti-pan neurofascin-associated neuropathy	(13)
Cerebellar disease	(5)
CIDP	(14)
Guillian Barré Syndrome	(15)
Multifocal motor neuropathy	(16)
Peripheral neuropathy	(1)
Pontocerebellar degeneration	(17)
Subacute nodopathy	(3)
Vision impairment	(5)
Multiple sclerosis	(8)

CIDP, chronic inflammatory demyelinating polyneuropathy.

### 2.2. Case 2

A 66-year-old man presented complaining of memory problems for the past two years. His medical history included nicotine dependency, hyperlipidemia, presbyacusis and post prostate cancer. Psychopathology was indicative of recurrent major depression, moderate (ICD-10: F33.1, Beck Depression inventory (BDI-II) score 31, i.e., depressive symptoms of severe severity). He is married, living with his wife, and has a daughter. He worked as a professional mason. He completed eight years of school and has a secondary school diploma, and has been retired for 3 years. His mother has severe dementia at an age of 87 years and requires constant health supervision. His father died of a myocardial infarction at the age of 46 years. At the time-point of neuropsychological testing, he reported Cognitive screening resulted in mild cognitive impairment (MMST 26/30). At neuropsychological testing, mild difficulties were detected in confrontation naming (language), and more severe cognitive deficits became apparent in information processing speed (attention), phonematic word fluency (language/executive functions), cognitive flexibility (executive functions), visuospatial cognition, working memory, visual memory and partly in verbal memory (Figure 1). His cognitive performance profile was classified as MCI in multiple domains together with mildly reduced ADL-competency (B-ADL: 4.4). His MRI revealed cerebral microangiopathy, but his brain fluorodesoxyglucose positron emission tomography (FDG-PET) yielded pathological Z-scores in the right prefrontal cortex, in the parietal inferior cortex on the right side, lateral occipital cortex on both sides, visual cortex on both sides, and temporal lateral cortex on the right side. Anti-neurofascin 186 autoantibodies were detected in his serum (1: 320) and CSF 1: 32 via anti-neural antigen IgG immunofluorescence testing with BIOCHIP-mosaic with brain tissue and recombinant cells. The neurofascin 186 autoantibodies were still present six months later (1:32). At follow-up he showed speech anomalies primarily entailing a stutter. However, he claims to have already stuttered when young, so that it is not clear whether this should be interpreted as a speech disorder or reactivation. The cognitive disturbances did not appear to be significantly progressive in his follow-up examination. He also denies suffering from hypomimia, vigilance fluctuations, REM sleep disturbances, and hallucinations.

## 3. Discussion

Our main finding here is the novelty of CNS involvement in neurofascin 186 antibody-associated autoimmunity over a follow-up of six months in two paradigmatic patients. Neurofascin 186 interacts with Neuropilin-1, which mediates axon guidance and adhesion during the formation of gamma amino butyric (GABA)ergic synapses in the cerebellum (6). Neurofascin 186 antibodies might have an impact on the function of GABAergic synapses in the cerebellum. Dysfunctional cerebellar GABAergic synapses might in turn affect cognitive functions via functional and anatomical connections between the cerebellum and hippocampus (7) as a potential mechanism of action of how neurofascin 186 antibodies might act. Another mechanism of action is based on axonal pathology with complement deposition induced by neurofascin 155 and 186 antibodies, which selectively target the nodes of Ranvier in multiple sclerosis (8). Other reports support the role of neurofascin autoantibodies in demyelinating diseases such as multiple sclerosis (9). Neurofascin antibodies appear to be much more common in primary progressive multiple sclerosis than in relapsing-remitting multiple sclerosis (10) (Table 1). These studies suggest that axonal pathology associated with neurofascin 186 autoantibodies may contribute to the progressive course. In one of our patients, we also detected elevated ptau181, suggesting axonal brain damage. Considering the mechanistic studies of how neurofascin 186 autoantibodies might contribute to axonal brain pathology, the ptau181 elevation in one patient can be partially explained. However, axonal pathology in our cases was not caused by multiple sclerosis. The neuroaxonal CNS damage in strategically cognitively relevant areas may contribute to cognitive dysfunction. Mild cognitive dysfunction could also coincide with multiple sclerosis, but further progression to dementia would be entirely atypical for multiple sclerosis. However, the exact mechanism of cognitive dysfunction in association with neurofascin 186 autoantibodies remains unclear in our patients, especially when considering the Bradford-Hill criteria (11). In our patient 2, it seems unlikely that dysimmune neuropathy and a combined central and peripheral demyelinating syndrome cause the neurofascin 186-associated cognitive dysfunction because of increased brain injury proteins and evidence of intrathecal IgG synthesis suggesting a central inflammatory process. Note that FDG-PET results demonstrate hypometabolism of the frontal, temporal, and parietal lobes in the second patient. Frontotemporal degeneration might therefore be the probable cause of cognitive dysfunction in patient 2, although clinical features for FTD behavioral variant or primary progressive aphasia were not met. In addition, the cognitive impairment began at age 64 years, suggesting possible disease from frontotemporal lobar degeneration. However, the clinical and neuropsychological profiles do not suggest FTD in the second patient.

Our follow-up investigations showed persistent neurofascin 186 autoimmunity only in one of the two patients; a peripheral nervous system affectation is also clinically conceivable with persisting neurofascin 186 autoantibodies. Although not formally retested, cognitive impairment was still obvious at follow-up and in both cases. We do not finally know whether autoantibodies against neurofascin 186 play a causal role in cognitive impairment in these two patients. Additionally, both patients suffered from major depression and were positive for cardiovascular risk factors, the latter most probably having caused cerebral microangiopathy found in both MRIs. This might in turn have contributed – at least in parts – to the observed cognitive impairment. Moreover and most interestingly, both patients had a positive family history for dementia. As patient 1 suffered peripheral nerve damage, the presence of neurofascin 186 antibodies should be regarded in conjunction with her peripheral nerve system affectation while considering the main manifestations of neurofascin 186-related disease described so far (Table 2: overview of neurofascin 186 antibody-related disease).

**TABLE 2:** Clinical and laboratory characteristics of patients.

Parameter	Patient 1	Patient 2
**Demographic parameters**		
Gender	Female	Male
Age in years	75	66
**Psychopathology**		
Disorientation	No	No
Attentional dysfunction	No	No
Memory disturbances	Yes	Yes
Formal thought disorder	No	Yes
Affective disturbance	Yes	Yes
Drive and psychomotor disturbance	Yes	Yes
**CSF**		
Cell count (<5μg/L)	1	3
Albumin mg/L	229	226
Tau protein (<450pg/ml)	163	128
P Tau protein 181 (<61pg/ml)	91	49
Aß42 (>450pg/ml)	2,185	1,082
Aß40	16,403	9,982
Ratio Aß42/40 × 10 (>0.5)	1.3	1.1
Blood brain barrier disturbance	No	No
Intrathecal IgG synthesis	Yes	No
**MRI**		
Generalized atrophy	Yes	No
Focal atrophy	No	No
Hippocampal atrophy	No	No
Vascular pathology	Yes	Yes

Aß42, Amyloid-ß 42; Aß40, amyloid-ß 40; CSF, cerebrospinal fluid; MRI, magnetic resonance imaging; P Tau Protein 181, phosphorylated tau protein 181; ratio Aß42/40, ratio of amyloid-ß 42/ratio of amyloid-ß 40; y, years. For laboratory data normal ranges are shown in brackets. Reference values: refers to reference values from the Neurochemistry Laboratory, Neurology Department, University Medical Center Göttingen.

## 4. Limitations

The main limitation of our case report is that we had no neuropsychological follow-up in either patient. In addition, the origin of MCI in both patients could also be influenced by vascular pathology, as evidenced by cerebral microangiopathy on MRI and the neuropsychological profile showing impairments in several cognitive domains. However, the mild degree of microangiopathy argue against a pronounced vascular pathology as a cause of MCI in these patients. In addition we cannot completely rule out that neurofascin 186 antibodies were false positive in the first case, as these were not replicated six months later. However, the clinically evident severe cognitive impairment and circumstantial evidence for neuroaxonal cell damage in the brain as well as intrathecal IgG synthesis support the possible role of these neurofascin 186 autoantibodies according to the recently published criteria for autoimmune-based psychiatric syndromes (12). It would be beneficial to measure ptau181 levels in a future study to see whether tau levels normalize over the time course of neurofascin 186-associated cognitive impairment, which we would expect to be the case in autoimmune-mediated cognitive impairment. In the second case, the repeated findings of neurofascin 186 antibodies in addition to brain abnormalities also argue against a false-positive effect of the autoantibody results. Another point worth mentioning is that no patient so far underwent immunotherapy as an individual drug trial, so the we cannot assess any benefit of immunotherapy which would have delivered potential evidence of a possible link between the cognitive impairment and an autoimmune basis of the neurofascin 186 autoantibodies.

## 5. Conclusion

Our results demonstrate that neurofascin 186 autoantibodies associated with amnestic MCI occur in multiple domains that should be considered in the differential diagnosis for mild cognitive impairment. The strength of this case series is the careful differential diagnosis with a large panel of neural autoantibodies, especially neurofascin 186 autoantibodies, and the longitudinal follow-up in both patients with MCI. Follow-up in both patients with stable cognitive impairment is also suggestive of an autoimmune-mediated time course and reveals no obviously progressive clinical course as in neurodegenerative diseases. These patients have not undergone immunotherapy because of the lack of evidence. More research is needed to investigate the presence of these autoantibodies in association with cognitive impairment in a larger homogeneous cohort without peripheral nervous system involvement and to include comprehensive clinical follow-up to better assess the clinical significance of autoantibody findings.

## Data availability statement

The raw data supporting the conclusions of this article will be made available by the corresponding author, without undue reservation.

## Ethics statement

Ethical approval is given for this study. The patients provided their written informed consent to participate in the study. Written informed consent was obtained from the individuals for the publication of any potentially identifiable images or data included in this study.

## Author contributions

NH and BC wrote the manuscript. All authors revised the manuscript for important intellectual content.
